# From across the Seas

**Published:** 1919-03-22

**Authors:** 


### FROM ACROSS THE SEAS.

### Doctors' Qualifications Questioned.

The members of the British Medical Association in Tasmania evidently scrutinise with great care the qualifications of those who do not participate in their strike against what they consider to be unsatisfactory hospital conditions. In the House of Assembly, arising out of the discussion on the Hospitals Bill, the diploma held by Dr. Ratten, Surgeon-Superintendent of the Hobart General Hospital, has been the subject of some inquiry. The Premier, challenging certain statements that had been made, stated that the diploma in question would be recognised in any part of the Commonwealth, but Mr. Whitsitt alleged that, as Dr. Ratten held a diploma dated 1907 of Harvey College (America), and the American Medical Association had stated that the Harvey Medical College of Chicago had lost its Charter in 1902, and became extinct in 1905, the subject could hardly be closed without further inquiry. The Premier then stated that his opinion as to the diploma was based on the fact that the holder had obtained registration both in Tasmania and South Australia, and no question had arisen until Dr. Ratten came to the assistance of the Government by accepting his present position. He would ask the House if they were going to ask Dr. Ratten to be placed at the mercy of men "who would crucify him if they got a chance, simply because he had helped the Government against the strike of doctors." The leader of the Opposition retorted that the most important point was the authenticity of the diploma, and that, in the interest of Dr. Ratten himself, called for investigation.

The upshot of the matter was that the Government appointed a Royal Commission composed of a Judge of the High Court, who examined the facts and reported in favour of the regularity of Dr. Ratten's registration. The medical Press strongly criticise the methods of inquiry adopted by the Commission.

Meanwhile the deadlock between the State Government and the British Medical Association, which is chiefly in respect of the treatment of rich patients in hospitals, still drags on.

### Medical Imposters.

From time to time some evil-disposed unqualified person succeeds in posing as a medical man and is detected and punished sooner or later. In Victoria an ingenious imposter recently successfully obtained employment as medical officer to a medical institute and had commenced practice. Prior to this he had had the effrontery to apply for registration, submitting a diploma of medical and surgical degrees purporting to issue from the Senate of the Royal University of Ireland. This document, forged from beginning to end, was a work of art and apparently presented the signatures of several officials with high-sounding names. Being compared with another diploma of the same University (now the National University of Ireland, Dublin) the Registrar was communicated with and the fraud discovered.

In England there was, a few months ago, a similar case, in which the culprit obtained work at a Red Cross Hospital, using the papers of a deceased practitioner, and was awarded the O.B.E. His fraud was later discovered and he fell from his glory.

